# Erratum: Lentiviral vectors can be used for full-length dystrophin gene therapy

**DOI:** 10.1038/srep46880

**Published:** 2017-08-29

**Authors:** John R. Counsell, Zeinab Asgarian, Jinhong Meng, Veronica Ferrer, Conrad A. Vink, Steven J. Howe, Simon N. Waddington, Adrian J. Thrasher, Francesco Muntoni, Jennifer E. Morgan, Olivier Danos

**Keywords:** Genetic vectors, Muscle stem cells

10.1038/srep44775

This article was published twice in error during a change in production systems. The publisher apologizes to the authors and readers for the error. When citing this work, please refer to the original version.^1^
